# Genetic Determinants of Osteoporosis: Common Bases to Cardiovascular Diseases?

**DOI:** 10.4061/2010/394579

**Published:** 2010-03-25

**Authors:** Francesca Marini, Maria Luisa Brandi

**Affiliations:** Department of Internal Medicine, University of Florence, Viale Pieraccini, 6, 50139 Florence, Italy

## Abstract

Osteoporosis is the most common and serious age-related skeletal disorder, characterized by a low bone mass and bone microarchitectural deterioration, with a consequent increase in bone fragility and susceptibility to spontaneous fractures, and it represents a major worldwide health care problem with important implications for health care costs, morbidity and mortality. Today is well accepted that osteoporosis is a multifactorial disorder caused by the interaction between environment and genes that singularly exert modest effects on bone mass and other aspects of bone strength and fracture risk. The individuation of genetic factors responsible for osteoporosis predisposition and development is fundamental for the disease prevention and for the setting of novel therapies, before fracture occurrence. In the last decades the interest of the Scientific Community has been concentrated in the understanding the genetic bases of this disease but with controversial and/or inconclusive results. This review tries to summarize data on the most representative osteoporosis candidate genes. Moreover, since recently osteoporosis and cardiovascular diseases have shown to share common physiopathological mechanisms, this review also provides information on the current understanding of osteoporosis and cardiovascular diseases common genetic bases.

## 1. Introduction

Osteoporosis is the most common age-related skeletal chronic disorder, characterized by reduced bone mass, deterioration of bone micro-architecture and increased risk of low-trauma fractures. Fragility fractures are the endpoint of osteoporosis and represent the major cause of morbidity and mortality. With the constant growing age of population, not only in developed countries but also in South America, Asia, and Africa, osteoporosis is becoming more and more a worldwide major public health problem. Today over two hundreds millions people worldwide and the thirty per cent of all postmenopausal women in USA and Europe are affected by osteoporosis. At least 40% of all affected women and 15–30% of all affected men will suffer a fragility fracture during their lifetime. The individuation of factors responsible for osteoporosis predisposition and development is fundamental for disease prevention and for setting of novel therapies. 

According to the International Osteoporosis Foundation (IOF) guidelines, osteoporosis risk factors can be divided into two main classes: (1) modifiable risks that depend principally on lifestyle and nutrition habits and can be modified and (2) fixed risks that are innate and cannot be modified. Main osteoporosis risk factors are depicted in Table 1.

Today Scientific Community agrees that osteoporosis is a complex multifactorial disorder caused by the interaction between environmental factors and genes that singularly exert modest effects on bone metabolism and fracture risk. Studies on osteoporosis sibs and families demonstrated that genetic factors are responsible for about 60–85% of inter-individual variability of bone mineral density (BMD) [1, 2], and this effect appears to persist even in the late decades of life. BMD heritability varies between different skeletal sites [3]. Also fragility fracture risk seems to have a genetic component; family history of fracture has been shown in some epidemiological studies as a risk factor for fractures [4, 5]. Interestingly, the heritability of fractures has been shown to be independent of BMD and maybe influenced by other factors such as bone geometry, bone turnover or the risk of falling. However, the heritability of fractures seems to decrease with age maybe as environmental factors become more important. Other bone features such as quantitative ultrasound properties, femoral neck geometry, bone turnover markers range have been demonstrated to be under the control of genetic factors [6, 7]. Except for some rare Mendelian monogenic inherited osteoporosis forms (osteoporosis associated with estrogen deficiency due to inactivating mutation of the aromatase gene (*CYP19*) [8] or associated with estrogen resistance due to inactivating mutation of the estrogen receptor alpha (*ER* *α*) gene [9], and autosomal recessive osteoporosis pseudoglioma caused by inactivating mutation of lipoprotein receptor-related protein 5 (*LRP5*) [10], classic age-related osteoporosis is a multifactorial heterogeneous disorder and, to date, its exact genetic bases are still unknown. In fact, bone metabolism is regulated by several genes, some exerting a high degree of influence (major genes) and other, even more numerous, exerting minor effects (minor genes). Due to the complex biology of the skeleton, putative osteoporosis candidate genes are very numerous and the number of genes identified to be involved in bone metabolism is constantly increasing. They include genes involved in the regulation of bone and calcium metabolism, such as those encoding for calciotrophic and sex hormones and their receptors, for bone matrix proteins, for cytokines, growth factors and local mediators and their receptors, and for proteins involved in molecular pathways of bone cells. Principal osteoporosis candidate genes are described in Table 2.

Moreover, the presence of epigenetic regulative factors, gene-gene and gene-environment interactions complicate the situation. Different genetic and environmental factors may result in the same osteoporotic phenotype and it is also possible that some individuals having one or more predisposing alleles and genetically at risk of osteoporosis never become osteoporotic or, controversially, individuals with no predisposing alleles may develop osteoporosis with age due to non-genetic factors. 

Candidate gene association studies have identified several polymorphisms associated to BMD, bone characteristics and fragility fracture risk. However, these association studies generated conflicting results may be due to inadequate population sampling, ethnicity, gender, age, confounding factors, lack of standardized genotyping methods, gene-gene interactions, linkage disequilibrium with other trait-causing polymorphisms in a nearby locus, epigenetic and/or post-transcriptional gene expression regulation (i.e., microRNAs) and gene-environment interactions. Retrospective meta-analyses, including several different association studies, and multicentric studies on large and well characterized populations are both helpful in reducing these issues and increasing the power of statistical associations. Several reviews on genetics of osteoporosis tried to summarize the most representative osteoporosis association studies [11–15]. 

Some of the most important and most studied osteoporosis candidate genes will be briefly discussed below, with specific focus on the results of the European Genomos study. The Genomos (Genetic Markers for Osteoporosis) study is an European multicentric consortium that collected over 20 000 Caucasian subjects (women and men) from several European centers, using prospective genotyping with cross-center standardization, for the study of osteoporosis candidate genes. 

For years osteoporosis and cardiovascular diseases (CVDs) have been considered as two independent consequences of aging, however, recent evidences support an association between these two diseases, indicating common physiopathological mechanisms, and maybe genetic bases.

## 2. Osteoporosis and Cardiovascular Diseases

Some studies have reported associations between age-related CVDs and bone loss and have indicated common etiologies for CVDs and osteoporotic fractures with a substantially increased risk of hip fractures in women after the diagnosis of a CVD [16–19]. 

More than 90% of atherosclerosis fatty plaques undergo calcification. Now it is well assessed that calcium metabolism has a central role both in bone mineralization and on the risk of arteriosclerosis development and progression, since regulatory factors of bone cells functions can also affect vascular calcification. Osteoprotegerin (OPG) and receptor activator of nuclear factor kappa B ligand (RANKL) regulate osteoclast activation and function but are also involved in the vascular calcification process and atherosclerosis [20, 21]. Bone morphogenetic protein (BMP2) is involved in osteoblastic differentiation by the stimulation of Runx2 expression; in humans, atherosclerotic lesions show an increased expression of BMP2 and Runx2 with respect to normal arteries [22] and this may be responsible for arteries wall calcification. Some others biological and environmental factors seem to be involved both in altered bone mineralization and in vascular calcifications, such as vitamin D insufficiency, low calcium intake, estrogen deficiency, chronic inflammation, oxidative stress, dyslipidemia, high dietary fat intake, smoking, low physical activity. Elderly people present with calcium and vitamin D deficiency that could contribute to calcium mobilization from bones with consequently higher risk of fractures and of severe vessels and arteries calcification. At the same time the age-related estrogen deficiency may induce the increase of pro-inflammatory cytokines (IL1, IL6 and TNF*α*) that enhances the expression of adhesion molecules on leukocytes and endothelial cells favoring the progression of atherosclerosis plaques. Estrogen deficiency also induces the decrease of OPG with subsequent calcium mobilization from bones and risk of calcification of atherosclerosis plaques. Last, estrogen deficiency induces a reduction of production of nitric oxide which has athero-protective effects but also plays a role in osteoblast function and regulates the endothelial function of bone microcirculation. Moreover, elevated LDL and low HDL cholesterol, suspected to be responsible for atherosclerosis, are associated also with low BMD and with vertebral fractures in postmenopausal women [23]. The altered lipid metabolism is associated with both bone remodeling and atherosclerosis process [22] and this may explain, in part, the coexistence of atherosclerosis and osteoporosis in patients with dyslipidemia. 

Animal, clinical and epidemiological studies suggest that high blood pressure is associated with abnormalities of calcium metabolism, leading to increased calcium loss, increased movement of calcium from bone and long-term risk of bone demineralization and osteoporosis [24–26]. Metabolic studies in hypertensive rats showed that associated hypercalciuria and secondary activation of parathyroid glands induced a reduced growth and a decreased bone mineral content later in life [27, 28]. The mechanisms by which this occurs are probably due to a defect in the kidney ability to handle calcium. Moreover, cross-sectional studies [29, 30] in humans have shown an inverse positive association between blood pressure and bone mineral density, supporting a possible correlation between hypertension and osteoporosis. 

Last, recent evidences of the action of bone antiresorptive drugs also on the reduction of CVDs risk and evidences of the positive effect of statins, antihypertensive drugs and insulin on bone mass increase [22, 31–33] suggest that osteoporosis and CVDs share common physiopathological molecular pathways. Bisphosphonates are potent antiresorptive agents widely used in osteoporosis treatment and in prevention of fracture risk. Experimental studies on animal models demonstrated that bisphosphonates also inhibit arterial and cardiac calcification in mice [34] and prevent foam cell formation by inhibiting LDL uptake and macrophages replication [33]. Raloxifene is a selective estrogen receptor modulator prescribed for both the prevention and treatment of osteoporosis and with a proven efficacy in the reduction of risk of fragility fractures. Raloxifene seems to have favorable effects on LDL cholesterol level and risk of coronary heart disease and it improves vascular endothelial function in postmenopausal women [35, 36]. The results from the MORE (Multiple Outcomes of Raloxifene Evaluation) randomized trial made possible to decipher the effects of raloxifene on cardiovascular events in osteoporotic postmenopausal women, evidencing that raloxifene therapy for 4 years did not significantly affect the risk of cardiovascular events in the overall studied population but it significantly reduced this risk in the subpopulation of women with increased cardiovascular risk [37]. Statins reduce cardiovascular mortality through the regression of coronary calcification and the reduction of LDL cholesterol levels in patients with dyslipidemia. These hypolipidemic drugs have also been associated to increased bone mineralization in mice [38] and in patients with osteoporosis [39] and with a reduction of fracture incidence. Last, recent clinical studies indicated that beta blockers and antihypertension drugs would reduce the risk of fragility fractures in the elderly population [40]. Moreover, in a rodent model angiotensin II type 1 and 2 receptor blockers (ARB) widely used antihypertensive agents, were shown to enhance bone mass through both the increase of osteoblast activity and the suppression of osteoclast activity [41]. Recently, a preventive effect of angiotensin II type 1 receptor blocker on osteoporosis has been reported but these data need confirmation [42]. The fact that all these drugs are effective on both osteoporosis and CVDs suggests a possible link between vascular and skeletal systems. Therefore, it is priority to establish to what extent treatments for osteoporosis are effective and beneficial for CVDs and vice versa, as well as to comprehend the exact physiopathological mechanisms shared by these diseases. 

According to the current knowledge, further specific studies are necessary to better define the relationship between osteoporosis and CVDs and to identify common risk factors and genetic determinants.

## 3. Vitamin D Receptor Gene (*VDR*)

Since the important role of vitamin D in the regulation of calcium homeostasis and bone metabolism, vitamin D receptor (*VDR*) gene has been the first candidate gene to be analyzed in association studies by Morrison at al. in 1994 [43] and it was proposed as a major locus for genetic effects on bone metabolism. Principal analyzed polymorphisms are the *BsmI*, *ApaI*, and *TaqI *polymorphisms in the 3′ UTR of the gene, the *FokI* polymorphism in exon 2 (that creates an alternative initiation traduction codon) and the *Cdx2* polymorphism in the promoter region of the gene. During the last two decades, several association studies have been performed but with conflicting data. Recent data seem to indicate that association between* VDR* polymophisms and bone mass is rather weak and the clinical impact of these variants remains unclear. Given the extent of published data on this gene and the non-concording results, interest now focuses on meta-analyses rather than single association studies alone. A haplotype meta-analysis by Thakkinstian et al. [44] evidenced that *VDR* single polymorphisms were not significantly associated to osteoporosis while *Bat* and *BAt *haplotypes were significantly associated, demonstrating the importance of haplotype studies rather than single polymorphism studies. 

The Genomos study collected 26,242 Caucasian participants (18,405 women) and evaluated the association between *Cdx2*, *FokI*, *BsmI*, *ApaI,* and *TaqI* polymorphisms with the DEXA-measure femoral neck and lumbar spine BMD and fractures, concluding that the *FokI*, *BsmI*, *ApaI,* and *TaqI *polymorphisms are not associated with BMD variation or with fractures while the “A” allele of the *Cdx2* polymorphism is associated with a reduced risk of vertebral fractures [45]. 

VDR is involved in vascular smooth muscle cell growth and in the regulation of calcium homeostasis and could therefore be involved in vascular plaques instability and calcification, thus, *VDR* polymorphisms may be associated with different risk for CVDs. In a study by Van Shooten et al. [46] the *bb* genotype of the *VDR* gene appeared to be predictive of severe coronary artery disease (CAD). The bb genotype is associated with low levels of circulating active form of vitamin D (calcitriol) [43], thus, results from this study seem to agree with previous finding of an inverse association between circulating calcitriol and CAD [47, 48]. The genetic association between VDR polymorphisms and CAD risk was not confirmed by a study in a Chinese population [49].

## 4. Estrogen Receptors Alpha and Beta Genes  (*ER*α** and *ER*β**)

Estrogens exert important effects on bone mass acquisition and maintenance. A rare case of a 28-year-old man with estrogen resistance and juvenile osteoporosis due to a nonsense inactivating point mutation in exon 2 of the *E* *R* *α* gene has been described [9]. Patient presented with unfused epiphyses and continuing linear growth in adulthood, indicating that estrogen is important for normal skeletal growth and bone development and mineralization in men as well as women. Genes encoding for estrogen receptors have been widely investigated in osteoporosis association studies. Most studies have focused on the (TA)_*n*_ repeat microsatellite in the promoter region and on the *PvuII* and* XbaI* single nucleotide polymorphisms (SNPs) in the intron 1 of the *E* *R* *α* gene. However, these studies generated conflicting results, suggesting the needing of large-scale investigations and analysis standardization. A meta-analysis by Ioannidis et al. [50] seemed to indicate no significant association between single *E* *R* *α* polymorphisms with BMD, while a significant reduction of fracture risk was associated to the *XX* genotype. Conversely, when *ER* *α* polymorphisms are analyzed together as haplotypes [51], significant associations of haplotypes with spinal BMD, decreased vertebral spine bone area and increased risk for spinal fracture were found in women, but not in men. This study also evidenced that *ER* *α*-dependent fracture risk is independent of BMD and bone area. 

The Genomos study collected 18 917 individuals (14,622 women) and evaluated the association of the three common *ER* *α* polymorphisms with the DEXA-measured femoral neck and lumbar spine BMD and with fractures [52]. The study evidenced that *ER* *α* polymorphisms exert only small effects on BMD. Conversely, the *XX *genotype resulted to be associated with a 20% reduction in fracture risk by mechanisms independent of BMD. 

However, to date most association studies on *ER* *α* gene have evaluated the association of its polymorphisms with BMD and fracture risk but not with bone structural and geometric properties. Recently, Cepollaro et al. [53] have evaluated the influence of *XbaI* and *PvuII *polymorphisms on structural and geometric bone parameters assessed by pQCT at the tibia in 541 Italian women and 449 Italian men. The study evidenced a significant association between the *PP* genotype of *PvuII* and higher values of tibial cortical thickness in male subjects. This result indicated a role for *E* *R* *α* gene in the control of tibia bone geometry and could explain the mechanism by which *ER* *α* gene polymorphisms influence fracture risk independently of BMD. 

*ER* *β* gene has been less studied in osteoporosis association studies since its role on bone metabolism is not yet completely known. *ER* *β* seems to have a role in mediating estrogen effect on bone growth and bone size but not on BMD [54]. A (CA)_*n*_ repeat microsatellite polymorphism in the promoter region has been associated with BMD in Asian [55, 56] and Caucasian [57] populations. No meta-analysis of *ER* *β* association studies is available so far. 

Estrogens have vasodilatatory, antiinflammatory and antiproliferative effects on the cardiovascular system and they have been reported to provide protection against CAD in postmenopausal women [58]. Lu et al. [59] associated the novel −1989T/G polymorphism in the *E* *R* *α* promoter B with CAD risk, concluding that the G/G genotype may be an independent predictor for CAD in patients with familiar hypercholesterolemia. In addition, they found that the long number (>17) of (TA)_*n*_ repeat in the *ER* *α* promoter region was significantly higher in postmenopausal women with CAD than in those without CAD, but not in men. Pollak et al. [60] confirmed this result and found an association between homozygote genotype for long alleles (>18) and a significantly higher angiographic severity of CAD in young patients. Alevizaki et al. [61] found that *E* *R* *α* *PuII* and *XbaI* polymorphisms may influence the severity of CAD in women, associating the C allele of *PvuII *and the G allele of *XbaI* with a higher number of arteries with a significant stenosis in the coronary angiography.

## 5. Collagen Type I *α*1 Gene (*COLIA1*)

COLI1A1 gene encodes for the *α*1 chain of collagen type I that is the principal proteic component of bone extracellular matrix, thus, this gene is an important candidate for osteoporosis risk. Several association studies have been conducted on a polymorphism in intron 1, a G/T substitution that creates a binding site for the transcription factor *Sp1*. The *s* *s* (T/T) genotype has been associated with reduced BMD [62, 63], increased age-related bone loss [64, 65], increased femoral neck angle [66], an impaired ability of osteoblast-like cells to form mineralized bone nodules in vitro and with abnormalities of bone mineralization in vivo [67] and a higher risk of fracture due to altered bone density and quality [68]. In general, association studies on this gene demonstrated a positive correlation between *SS* (G/G) genotype and reduced fracture risk even with lack of association with BMD values. A meta-analysis evidenced that different *Sp1* alleles are associated with modest variation in BMD but with significant changes in fracture risk [69]. The T allele is associated with an abnormally increased synthesis of collagen I *α*1 chain generating an imbalance between the *α*1 and *α*2 chains and a reduction of bone strength and bone matrix mineralization [67].

The Genomos study collected standardized data on 20,786 individuals and evaluated association of the *Sp1* polymorphism with the DEXA-measured femoral neck and lumbar spine BMD and with fractures [70]. The *s* allele resulted to be associated with recessive-inherited reduced BMD. However, this association was quite weak. Authors also found a modest association of the *s* allele with vertebral fractures, particularly in women, and hypothesized that this allele could predispose to incidental vertebral fractures independently of BMD.

## 6. Transforming Growth Factor *β*1 (*TGF*β*1*)

*T* *G* *F* *β* *1*gene encodes a local growth factor that is widely expressed by bone cells and it is involved in the regulation of bone turnover [71–73]. This gene has been analyzed as a possible osteoporosis candidate gene; principal investigated polymorphisms are located in the promoter region (−*1348C/T *and −*509C/T*) and in the exon 1 (*29T/C Leu10Pro* and *74G/C Arg25Pro*). Several studies have associated variants in this gene with BMD variation and/or fragility fractures risk [74–80], but none of them have investigated the effects of *TGF* *β* *1* haplotypes. 

The Genomos study performed a wide standardized analysis including 28 924 participants from 10 European centers [81]. The study genotyped five polymorphisms of *TGF* *β* *1 *gene (two located in the promoter region, two located in the exon 1 and one located in exon 5) and associated them with DEXA-measured BMD of lumbar spine and femoral neck and with fractures. None of the polymorphisms or haplotypes resulted to be associated to BMD variations or to affect the overall risk of fractures. A weak association was detected between carriers of the rare *788T* allele of the *788 C/T* polymorphism (*Thr263Ile*) in exon 5 and the risk of incident vertebral fractures. 

TGF*β* seems to have contrasting functions on cardiovascular system. Some studies reported a protective effect of TGF*β* by reducing the risk of CVDs [82–85], while others described TGF*β* as inducing or facilitating CVDs such as vascular stenosis and thrombogenesis [86, 87]. The *Arg25Pro* polymorphism in exon 1 has been associated with different risk of essential hypertension in Russian male individuals [88], with variation in systemic blood pressure in essential hypertensive patients [89] and with different risk of myocardial infarction or hypertension in Caucasian patients [90], but no correlation has been found between this polymorphism and the risk of myocardial infarction and stroke [91], the risk of CAD in Caucasian patients [92] and with the severity of CAD and the occurrence of myocardial infarction or hypertension in Australian patients [93]. The Leu10Pro polymorphism in exon 1 has been associated with susceptibility to myocardial infarction in a Japanese population [94], with clinical characteristics of hypertension [95] and with the risk of stroke in an elderly Caucasian population [91], but not with the risk of myocardial infarction in the same elderly Caucasian population [91].

## 7. Low-Density Lipoprotein Receptor-Related Protein 5 and 6 (*LRP5* and *LRP6*)

*LRP5* and its related homologue *LRP6 *are cell-membrane coreceptors for Wnt proteins an the Wnt/beta catenin signalin pathway, that controls osteoblast activity and bone formation. *LRP5* gene has a clear role in rare bone diseases and also in normal variation in peak BMD.

 Inactivating mutations of *LRP5* lead to osteoporosis pseudoglyoma [96] while activating mutations result in high bone mass phenotypes [97, 98]. 

Point mutations in LRP6 gene cause abnormal formation of the axial skeleton and a low bone mass phenotype in mice [99]. 

Thus, common variants in *LRP5* and *LRP6* genes may contribute to normal population variance in human bone metabolism, and these two genes have been recently proposed and analyzed as putative osteoporosis candidate genes. Two studies [100, 101] have demonstrated an association between *LRP5* variants and bone mass in human populations. Particularly, Ferrari et al. [100] found an association between *2047 G/A* substitution (*Val667Met*) in exon 9 and bone mineral content at lumbar spine, bone area and with stature in men, but not in women. 

A study by van Meurs et al. [102] analyzed the role of four variants of the *LRP5* gene and one aminoacid variant of the *LRP6* gene in determining BMD, bone geometry and fracture risk. Authors found that the* 1330Val* allele (*Ala1330Val* polymorphism) of the* LRP5* gene was associated with a decreased BMD at lumbar spine and femoral neck and with reduced vertebral body size and femoral neck width in men. Male carriers of the *1330Val* allele had a 60% increased risk for fragility fractures. A borderline association of the *LRP6 Ile1062Val* polymorphism with height and vertebral body size was observed in men. Males carriers of the *1062Val* allele had a 60% higher risk of fractures. In women all these association were weaker than in men. 

All these studies indicated that *LRP5* and *LRP6* associations with bone phenotypes are sex specific.

The Genomos study collected and analyzed 37,534 Caucasian individuals from Europe and North America and associated DEXA-measured BMD at lumbar spine and femoral neck and fracture risk with *Val667Met* and *Ala1330Val* polymorphisms of the *LRP5* gene and with *Ile1062Val* polymorphism of the *LRP6* gene [103]. The *Met667* and *Val1330* alleles were both associated with reduced spinal and femoral BMD and with increased risk of vertebral or total fractures. Haplotype analysis indicated that *Met667 *and *Val1330 *variants affected BMD independently. Conversely, the *LRP6 Ile1062Val* polymorphism was not associated with any osteoporotic phenotype. 

No studies on the association of *LRP5* and *LRP6* polymorphisms with CVDs risk are available so far.

## 8. Aromatase Gene (*CYP19A1*)

Aromatase enzyme catalyses the conversion of androgens to estrogens. Inactivating mutations of the *CYP19A1* gene have been associated, in both sexes, with an increased bone turnover and consequently with a decreased BMD [8]. 

A microsatellite tetranucleotide (TTTA)_*n*_ repeat polymorphism in intron 4 was associated to osteoporosis risk [104]. Analysis of polymorphism in a postmenopausal cohort of Italian women associated the (TTTA)_12_ allele with a protective action versus osteoporosis development. Women with high number of repeats (>11) showed higher lumbar BMD values than women with low number of repeats (from 8 to 11). However, the molecular mechanism that can explain association between aromatase activity and (TTTA)_*n*_ repeat is still unknown. 

Aromatase has been hypothesized as one of the factor affecting blood pressure and maybe a susceptibility gene for hypertension. A study by Shimodaira et al. [105] found an association between the rs700518 and rs10046 polymorphism, as well as a haplotype constructed with rs1870049 and rs10046 polymorphisms, of *CYP19A1* gene with variation in systolic blood pressure and diastolic blood pressure. Interestingly, the at risk genotypes of rs700518 and rs10046 showed a sex-dependent inverse relationship, suggesting the possibility to use them as genetic markers for gender-specific essential hypertension risk. Ramirez-lorca et al. [106] confirmed that the rs10046 polymorphism may be involved in the genetic regulation of blood pressure in women. Another study by Letonja et al. [107] concluded that in Caucasian subjects the (TTTA)_*n*_ repeat polymorphism does not contribute to the genetic susceptibility to CAD.

## 9. Insulin-Like Growth Factor I Gene (IGF-1)

IGF-1 exerts anabolic effects on BMD increasing synthesis of collagen type I and osteocalcin and stimulating alkaline phosphatase activity that results on both proliferation and differentiation of osteoblasts. It is an essential factor during childhood and adulthood in the regulation of trabecular and cortical bone formation. 

Association studies evaluated a (CA)_*n*_ repeat in the promoter region of the gene but with controversial results. The presence of the 194-bp allele has been associated to higher BMD and increased level of circulating IGF-1 in osteoporotic Korean women with respect to healthy controls [108]. Low levels of IGF-1 and a reduced BMD were associated to the homozygote 192 bp allele in men with idiopatic osteoporosis [109] and in Caucasian postmenopausal women [110]. However, a study on Japanese postmenopausal women [111], a study on premenopausal Chinese women [112], and a study on premenopausal Caucasian and Afro-American sibling pairs [113] did not confirm the precedent results.

## 10. Interleukin 6 (*IL6*)

After menopause the increase of IL6 and other pro-inflammatory cytokines production related to the estrogen decrease has been associated to the extent of bone loss [114]. The *IL6* gene locus (*7p21*) has been associated with BMD variations in postmenopausal women [115]. Two polymorphisms (−*572G/C* and −*174G/C*) in the promoter region of the *IL6* gene have been associated to markers of bone resorption in postmenopausal women [116, 117]. A study of Ferrari et al. [118] evidenced that the G/G genotype of the −*174G/C* polymorphism was associated to a lower BMD with respect to the G/C and C/C genotypes in over 15-year postmenopausal women but not in premenopausal women and in men. These results seem demonstrate that *IL6* polymorphisms regulate bone mass only after menopause with a molecular mechanism dependent on the estrogen deficiency. 

IL6 is a key mediator of inflammation and it has been demonstrated to play an important role in the pathogenesis of atherosclerosis and vascular diseases. Elevated concentrations of IL6 are predictive of future coronary events in healthy individuals and of mortality in patients with acute coronary disease. The −*174G/C *polymorphism in the promoter region has been associated with variation in IL6 production. Several studies have demonstrated that this polymorphism is associated with risk of coronary hearth disease in men [119–122], with the risk of ischemic carebrovascular events [123], with carotid artery compliance, systolic and diastolic blood pressure and serum high-density lipoprotein cholesterol in men [124], ischaemic stroke [125], number of severely stenosed coronary arteries [126]. However, despite of numerous studies the role of this polymorphism as a risk factor for CVDs remains inconsistent [127, 128] and needs for further researches.

## 11. Other Candidate Genes

Other osteoporosis candidate genes have been studied even if less extensively and with less conclusive results. They include calcitonin receptor (*CTR*) [129–133], calcium sensing receptor gene (*CaSR*) [134–136], androgen receptor (*AR*) [137], parathyroid hormone receptor (*PTHR1*) [138], sclerotin (*SOST*) [139], bone morphogenetic protein 2 (*BMP2*) [140], osteoprotegerin (*OPG*) [141, 142], and so forth. All these gene require confirmation on larger cohorts.

## 12. Genome-Wide Association Studies

The complete sequencing of human genome [143], the results of the HapMap project [144] and the development of novel chip technologies have opened novel avenues for the identification of genetic loci, genes and/or polymorphisms associated with complex diseases such as osteoporosis, through the application of genome-wide association analyses. This approach has permitted the simultaneous analysis of hundreds loci/genes along the human genome and the identification of novel osteoporosis subsceptibility loci and/or genes that were not candidates based on the current knowledge of the phatophysiology of bone metabolism and osteoporosis. Over 60 quantitative trait loci (QTLs) have been associated with bone metabolism and they were located in all but chromosome Y [145]. A list of genetic loci identified to be linked to bone metabolism through genome-wide approaches, and replicated in at least two different studies, are reported in Table 3. The first osteoporosis genome-wide association study [146] associated 100,000 SNPs with BMD values, bone ultrasound properties and hip geometry index. Some weak associations with genetic markers within or near known osteoporosis candidate genes (i.e., *E* *R* *α*, *CYP19*, *COLIA1,* and *LRP5*) were detected. 

Particularly, the application of genome-wide association scan has allowed the identification of bone morphogenetic protein 2 (*BMP2*) as a candidate gene for osteoporosis, through the analysis of 207 osteoporotic families (1323 individuals) in Iceland and the subsequent followup association analysis [140]. Recently, also the latent transforming growth factor beta binding protein 2 (*LTBP2*) [148] and the signal transducer and the activator of transcription 1 (*STAT1*) [149] genes have been associated with osteoporotic phenotypes. 

Given the extensive number of identified candidate loci to date, and given the potential large number of genes within these loci, the next step will be the refinement of the significant loci and the identification of putative candidate genes. Novel identified candidate genes have to be confirmed by follow-up population-based association studies and functional studies. Caution should be taken in the interpretation of replication/confirmation of the results since some genomic region could eventually be proven to be a false positive. 

The past few years have seen a significant increase in the number of genetic loci associated with CVDs through genome-wide association studies. Significant results have been reported in a recent review [147].

## 13. Animal Models

Comparative genetics is helpful in the comprehension of molecular mechanisms of bone remodeling and in the searching for osteoporosis candidate genes. Studies in animals are essential because they allow breeding strategies that cannot be performed in humans and they also provided extreme bone strength phenotypes that cannot be measured in vivo in humans. Rodents and primates are the most suitable models. Linkage studies in rats [150], mice [151, 152] and primates [153] have permitted the identification of numerous QTLs that regulate BMD and other bone quality properties (shape, microstructure and strength). Particularly, linkage analysis in mouse has allowed the identification of *Alox15* gene as a negative regulator of BMD. The *Alox15*-knock-out mice presented an increased BMD and the inhibition of *Alox15* expression compensated the ovariectomy-induced bone loss [154]. Recent studies have shown that genetic variations in a human homologue of *Alox15* (*ALOX12*) accounted for approximately 3% of the bone mass variation in humans [155, 156]. Moreover, the using of knock-out and transgenic animal models for the study of bone monogenic diseases has helped in the identification of biologically relevant osteoporosis candidate genes for association studies. These approaches also helped in delivering biological function of a specific gene in bone metabolism. An example is the *LRP5* gene whose activating or inactivating mutations have been demonstrated to be responsible for opposite extreme bone phenotypes [10, 96–98], using specific *LRP5*-mutated animal models.

## 14. Future Perspectives: the Pharmacogenetics of Osteoporosis

A very novel area of the genetics of osteoporosis is the pharmacogenetics of osteoporosis. Pharmacogenetics is the application of genetic studies to predict the outcome of drug treatments with respect to both beneficial and adverse events, and it is particularly important in chronic diseases, such as osteoporosis, that require long-term drug treatments and for which alternative effective drug therapies are available. Potentially pharmacogenetics of osteoporosis will allow clinicians to choose in advance the best treatment and the most effective drug regimen based on patient genotype. However, to date only few studies on the pharmacogenetics of osteoporosis have been published and no clinical applications are available. Pharmacogenetic studies associated osteoporosis candidate genes (*VDR,* *E* *R* *α*, *E* *R* *β*, and *COLIA1*) with the response to antiresorptive and antifracture agents such as hormone replacement therapy, raloxifene and bisphosphonates [157] and found weak associations. Recently a study by our Research Group [158] associated the *A/C rs2297480* polymorphism of the *FDPS *gene with the response to amino-bisphosphonates in a cohort of Danish osteoporotic women. 

All these studies suggested the possibility to use genetic screening to tailor decisions about osteoporosis antifracture treatment choice; however, all these preliminary data need to be confirmed and validated by large-scale studies, by prospective well-designed clinical trials and by functional analyses. 

Data from studies on pharmacogenetics of osteoporosis would be useful also in the field of CVDs since there are scientific evidences of the positive action of antiresorptive drugs, such as bisphosphonates and raloxifene, on the reduction of CVDs risk [22, 31–33].

## 15. Conclusions

The contribution of genetic factors to age-related chronic diseases such as osteoporosis and CVDs is important. The identification of genes that contribute to the pathogenesis of such disorders has potential public health, clinic and therapeutic implications. However, since osteoporosis is a complex multifactorial disease, the association studies performed to date presented with non-concluding or conflicting results. Now it is clear that a single SNP exerts less than 1–3% of effect in the determination of bone metabolism. Only large-scale standardized studies in well-characterized and homogenous populations and the study of haplotypes and/or multiple SNPs and/or genes might help in a better understanding of genetic factors underlying bone phenotypes heritability and calcium and lipid metabolism regulation. Moreover, technological advances such as genome-wide association scan will help in identifying novel candidate loci and/or genes, in validating the role of candidate gene polymorphisms and in analyzing hundreds of SNPs simultaneously. 

However, genetic epidemiology association studies did not tell how genes contribute to the disease, thus, functional genomic studies, large-scale gene expression studies and proteomic studies are fundamental for the comprehension of molecular and cellular mechanisms regulating bone and cardiovascular pathophysiology. The aim of functional genetics is not only to collect information about single gene functions but also to understand how biological component work together to regulate bone metabolism and cardiovascular system functionality. The great challenge of genetics of osteoporosis and cardiovascular diseases will be not only identifying the responsible genes but also understanding how these genes act and how they are influenced by other biological and/or environmental factors.

## Figures and Tables

**Table 1:** Main risk factors for osteoporosis.

Osteoporosis modifiable risks	Osteoporosis fixed risks
(i) Alchool	(i) Age
(ii) Smoking	(ii) Ethnicity
(iii) Low body mass index	(iii) Female gender
(iv) Poor nutrition	(iv) Family history of fractures
(v) Eating disorders	(v) Previous fractures
(vi) Insufficient physical activity	(vi) Menopause/hysterectomy
(vii) Low dietary calcium intake	(vii) Hormonal status
(viii) Vitamin D deficiency	(viii) Long-term glucocorticoid therapy
(ix) Frequent falls	(ix) Primary/secondary hypogonadism in men

**Table 2:** Main osteoporosis candidate genes.

Putative candidate genes	Chromosome location	Biological functions in bone and mineral metabolism	Main polymorphisms analyzed in osteoporosis association studies
Calciothropic and sex hormones and their receptors			
(i) Vitamin D receptor (*VDR) *	12q12-14	Calcium and phosphatase homeostasis; regulation of osteoclasts and osteoblasts functions.	*Cdx2* (promoter region); *FokI* (exon 2); *BsmI*, *ApaI*, *EcoRV* and *TaqI* (3′ UTR)
(ii) Parathyroid hormone (*PTH*) and PTH receptor (*PTHR*)	11p15 and 3p22-21	Calcium homeostasis, endogenous vitamin D synthesis and regulation of bone cells activity	*BstBI *(intron 2 of *PTH *gene); (AAAG)_*n*_ repeat (P3 promoter of *PTHR *gene)
(iii) Estrogen Receptor Alpha and Beta (*E* *R* *α* and *E* *R* *β*)	6q25 and 14q22-24	Control of bone remodelling, reduction of bone resorption	(TA)_n_ repeat (promoter region) and *XbaI* and *PvuII *(intron 1) (*E* *R* *α*); (CA)_*n*_ repeat (promoter region) and AluI (exon 8) (*E* *R* *β*)
(iv) Calcitonin (*CT*) and its receptor (*CTR*)	11p15 and 7q21	Increasing of osteoblast activity, retaining of calcium in bones and prevention of phosphorus and calcium loss	(CA)_*n*_ repeat (5′ flanking region of *CT* gene); *AluI *(nucleotide 1377 of *CTR* gene)
(v) Aromatase (*CYP19A1*)	15q21	Catalyzation of androgens conversion to estrogens	(TTTA)_*n*_ repeat (intron 4)
(vi) Androgen receptor (*AR*)	Xq11-12	Regulation of osteoblast function and suppressive action on bone resorption	(CAG)_*n*_ repeat (exon 1)
(vii) Calcium-sensing receptor (*CaSR*)	3q13-21	Regulation of calcium homeostasis at parathyroid, kidney, bowel and bone level	(CA)_*n*_ repeat (5′ flanking region at the *CaSR* locus); T/C Ala986Ser (exon 7)
(viii) Glucocorticoid receptor (*GR*)	5q31	Inhibition of bone formation, suppression of calcium absorption	Asp363Ser (exon 2)

Cytokine, growth factors and local regulators			
(i) Interleukin-6 (*IL6*)	7p21	Effect on osteoclastogenesis and bone resorption	(CA)_*n*_ repeat (5′ flanking region at the *IL6* locus); −634 C/G, −572G/C and −174G/C (promoter region).
(ii) Insulin-like growth factor 1 (*IGF-I*)	12q22-24	Stimulation of bone formation, recruitment of pre-osteoblasts, growth factor for osteoblasts	(CA)_*n*_ repeat (promoter region)
(iii) Transforming growth factor *β*1 (*TGF* *β* *-*1)	19q13	Osteoclast and osteoblast activity	−1348C/T and −509C/T (promoter region), 29T/C Leu10Pro and 74G/C Arg25Pro (exon 1), 869T/C and 788C/T Thr263Ile (exon 5), 713-8delC (intron 4), 861-20T/C (intron 5)
(iv) Bone morphogenetic protein 7 (*BMP7, OP1*)	20q13	Promotes mesenchymal cells into osteoblastic differentiation	—
(v) Bone morphogenetic protein 4 (*BMP4*)	14q22-q23	Involved in bone and cartilage development and in fracture repair	−5826G/A (promoter region); 3564C/T (intron 2); 6007C/T Ala147Val (exon 4)
(vi) Bone morphogenetic protein 2 (*BMP2*)	20p12	Stimulates the differentiation and/or activity of osteoclasts	Ser37Ala (exon 2); A/G Ser87Ser (exon 2); Arg190Ser (exon 3)

Bone matrix proteins			
(i) Collagen type I alpha1 (*COLIA1) *	17q21-22	Encode for collagen type I alpha1 chain	Sp1 (binding site of the transcription factor Sp1 in the intron 1)
(ii) Collagen type I alpha2 (*COLI-A2*)	7q22	Encode for collagen type I alpha2 chain	*PvuII*, *RsaI*, (ACT)_*n*_ repeat (intron 12)
(iii) Osteopontin (*OPN, SPP1*)	4q21-25	Anchoring osteoclasts to the mineral matrix of bones	Intragenic (CA)_*n*_ repeat
(iv) Osteocalcin (*OCN, BGLAP*)	1q25-q31	Role in bone matrix mineralization and calcium homeostasis	C/T and (CA)_*n*_ repeat (promoter region)
(v) Osteonectin (*ON, SPARC*)	5q31.3-q32	Binds calcium, initiates mineralization and promotes mineral crystal formation	Intragenic (CA)_*n*_ repeat

Miscellaneous			
(i) Low-density lipoprotein receptor-related protein 5 (*LRP5*)	11q13	Regulates osteoblasts proliferation and bone formation	Val667Ala (exon 9); Ala1330Val (exon 18)
(ii) Low-density lipoprotein receptor-related protein 6 (*LRP6*)	12p13.3-p11.2	Regulates osteoblasts proliferation and bone formation	Ile1062Val (exon 14)
(iii) Receptor activator of nuclear factor kappa B (*RANK*)	18q22.1	Receptor of RANKL expressed on osteoclast precursors and osteoclasts. The association of RANK-RANKL regulates osteoclast formation, activation and survival in normal bone modelling	421C/T, 575C/T
(iv) RANK ligand (*RANKL*)	13q14	Ligand of RANK expressed on osteoblasts and stromal cells. The association of RANK-RANKL regulates osteoclast formation, activation and survival in normal bone modelling	−290C/T, −643C/T, −693G/C and −1594G/A (promoter region)
(v) Osteoprotegerin (*OPG*)	8q24	Soluble “decoy receptor” of RANKL, inhibits osteoclast development by blocking the RANK-RANKL interaction	163A/G, 209G/A, 245T/G and 1181G/C (exon 1)
(vi) Sclerotin (*SOST*)	17q11.2	Potent osteocyte expressed negative regulator of bone formation in vitro	−9247T/C, −7859C/G, −1605C/T and −1396delGGA (promoter region); 27G/A (exon 1)
(vii) Chloride channel 7 (*CLCN7*)	16p13	Encodes a Chloride channel highly expressed in osteoclasts and essential for acidification of the resorption lacuna	Ala390Ala (exon 14), Val418Met (exon 15)
(viii) Methylentetrahydrofolate reductase (*MTHFR*)	1p36.3	Involved in collagen synthesis	677C/T (exon 4)

**Table 3:** QTLs identified and replicated in genome-wide linkage studies for osteoporosis [145]. Asterisks indicate genetic region associated also to clinical CVD events through genome-wide association studies [147].

QTLs replicated in at least two studies	QTLs replicated in at least three studies	QTLs replicated in at least five studies
- 2p21-24	- 13q31-34	- 1p36
- 2q33-37*	- 17p11-13	- 1q21-24
- 3q12-26		- 4q31-34
- 4p15-16		- 12q23-24*
- 5q33-35		
- 6p21		
- 8q24-qter		
- 10q26		
- 11q23-24		
- 14q12-24		
- 14q31-32		
- 16p13		
- 19p13-q13		
- 21q22-qter*		
